# The Influence of Pressure-Swing Conditioning Pre-Treatment of Cattle Manure on Methane Production

**DOI:** 10.3390/bioengineering7010006

**Published:** 2019-12-30

**Authors:** Britt Schumacher, Timo Zerback, Harald Wedwitschka, Sören Weinrich, Josephine Hofmann, Michael Nelles

**Affiliations:** 1 Biochemical Conversion, DBFZ Deutsches Biomasseforschungszentrum Gemeinnützige GmbH, Torgauer Straße 116, D-04347 Leipzig, Germany; Timo.Rolf.Zerback@dbfz.de (T.Z.); Harald.Wedwitschka@dbfz.de (H.W.); Soeren.Weinrich@dbfz.de (S.W.); Josephine.Hofmann@dbfz.de (J.H.); michael.nelles@uni-rostock.de (M.N.); 2 Department Waste and Resource Management, University of Rostock, Justus-von-Liebig Weg 6, D-18057 Rostock, Germany

**Keywords:** anaerobic digestion, cattle manure, steam explosion, pre-treatment

## Abstract

Cattle manure is an agricultural residue, which could be used as source to produce methane in order to substitute fossil fuels. Nevertheless, in practice the handling of this slowly degradable substrate during anaerobic digestion is challenging. In this study, the influence of the pre-treatment of cattle manure with pressure-swing conditioning (PSC) on the methane production was investigated. Six variants of PSC (combinations of duration 5 min, 30 min, 60 min and temperature 160 °C, 190 °C) were examined with regards to methane yield in batch tests. PSC of cattle manure showed a significant increase up to 109% in the methane yield compared to the untreated sample. Kinetic calculations proved also an enhancement of the degradation speed. One PSC-variant (190 °C/30 min) and untreated cattle manure were chosen for comparative fermentation tests in continuously stirred tank reactors (CSTR) in lab-scale with duplicates. In the continuous test a biogas production of 428 mL/g volatile solids (VS) (54.2% methane) for untreated manure was observed and of 456 mL/g VS (53.7% methane) for PSC-cattle-manure (190 °C/30 min). Significant tests were conducted for methane yields of all fermentation tests. Furthermore, other parameters such as furfural were investigated and discussed.

## Highlights:

- 109% methane yield increase through PSC-treatment of cattle manure in BMP-test;
- 160 °C/60 min and 190 °C/30 min are the best case of PSC-treatment in BMP-test;
- accelerated methane formation via PSC-treatment in BMP-test;
- temporarily enhanced methane production in semi-continuous test after treatment.

## 1. Introduction

Germany is the country leading by far in the primary production of biogas in European Union with 7852.4 ktoe in 2015, compared to United Kingdom with 2252.4 ktoe and Italy with 1871.5 ktoe biogasbarometer [1]. In 2016, approximately 8535 biogas plants fed with excrement and energy crops, biowaste or organic waste produced biogas in Germany (Daniel-Gromke, 2018) [2]. In contrast to other countries, the biogas is not only produced in landfills and sewage treatment plants in Germany [1], but mainly in the agricultural sector with energy crops and excrement as feedstock (Daniel-Gromke, 2018) [2]. Nevertheless, Germany has a high biogas production potential from manure with 90 PJ/year, which is not fully tapped (Scheftelowitz and Thrän, 2016) [3]. One of several reasons is the logistical challenge because the potentials are spread over a large number of farms (Scheftelowitz and Thrän, 2016) [3]. Another technical challenge is the handling of solid manure in practice and its slow degradation speed in anaerobic digestion. However, greenhouse gas (GHG) emissions could be saved by manure management via biogas generation as well as by substituting fossil fuels by biogas (Scheftelowitz and Thrän, 2016) [3].

Generally, bedding material in manure like straw consists to a large extent of ligno-cellulose. Hendriks and Zeeman (2009) [4] identified inter alia the crystallinity of cellulose, available surface area, and lignin content as limiting factors for the decomposition of ligno-cellulosic biomass. Hence, a pre-treatment of solid manure is advisable to improve the decomposition and to overcome technical challenges like floating layers in plug flow digesters or continuously stirred tank reactors (CSTR). In practice, physical pre-treatment methods are dominant, in 81% of German biogas plants (Schumacher, 2014) [5], but thermal pressure hydrolysis is a comparatively recent and seldom implemented process in the agricultural sector.

Lab-scale batch tests with cattle manure pretreated with thermal pressure hydrolysis revealed contradictory results. Risberg et al. [6] found no improved gas production, while Budde et al. [7] measured increased methane yields by up to 58% with a treatment temperature of 180 °C. Besides the positive effect of surface enlargement of a thermal pressure hydrolysis, negative effects may occur due to the formation of inhibiting substances. These substances include phenolic compounds as well as furalaldehydes, e.g., furfural and 5-hydroxymethylfurfural, which are the main degradation products derived from the dehydratation of hexoses or pentoses, respectively (Barakat et al., 2012) [8]. The negative effects of such by-products have been widely investigated for biotechnological ethanol or hydrogen production, whereas studies focusing on methane production all still very limited. One possible reason could be due to the fact that the microbial consortium involved in biogas production tends to have a higher tolerance to such inhibitory by-products (Monlau et al., 2014) [9]. For example, the treshold value for furfurals in biotechnological hydrogen production with single cultures is often given with 0.62 g/L (Siqueira and Reginatto, 2015) [10], whereas similar concentrations in biogas production generally lead to no negative effects (Pekařová et al., 2017) [11]. Further reasons could be due to lower substrate/inoculum and inhibitor/inoculum ratios as well as different incubation times (Monlau et al., 2014) [9]. Nevertheless, inhibitory impacts on biogas production could be observed especially at higher furfural concentrations too (Pekařová et al., 2017) [11]. For this reason concentrations of toxic by-products should always be taken into account during hydrothermal substrate pretreatment.

However, discontinuous biochemical methane potential (BMP) tests are limited to a basic assessment of methane yield, anaerobic biological degradation kinetics and qualitative assessment of inhibitory effects of the substrate (VDI 4630, p. 44) [12], Schumacher et al., 2019 [13]. For the assessment of process stability behavior, synergistic effects, mono-fermentation and/or limits of organic loading rate of substrates as cattle manure, continuous running anaerobic digestion (AD) tests have to be conducted (VDI 4630, p. 44) [12], Schumacher 2019 [13]. Nevertheless, continuous running AD tests have the disadvantage that they are time-consuming and labor-intensive. These are the reasons why studies about the effects of pre-treatment of cattle manure with thermal pressure hydrolysis in CSTRs are rare.

The aim of this study was to examine the influence of cattle manure’s pre-treatment with pressure-swing conditioning (PSC) under varying treatment conditions (treatment temperature, treatment duration) on the methane yield in batch tests. Additionally, a semi-continuous test in CSTRs in duplicates was conducted at lab-scale. The test were operated for 2.5 hydraulic retention times, in order to evaluate process stability during the feeding of a selected PSC-sample in comparison to the reference. These labor-intensive continuous tests are beyond the investigations of most other authors. Furthermore, PSC is a specific thermal pressure hydrolysis module in pilot-scale under development of the company VENTURY GmbH Energieanlagen (Dresden, Germany).

## 2. Materials and Methods

Cattle manure from a German farm was collected several times and pre-treated by the company VENTURY GmbH Energieanlagen (Dresden, Germany) for BMP tests as well as for semi-continuous tests in CSTRs. The cattle manure consisted of excrements from calves and cereal straw as bedding material.

### 2.1. Pre-Treatment and Biomethane Potential (BMP) Testing

The cattle manure (excrements and straw together) was treated with different combinations of temperatures and treatment times with PSC. The operation of the PSC pilot plant was described also by Schumacher et al. (2019) [13]. Approximately 5 kg of manure were fed manually into the pressure tank and heated with steam to the chosen temperature. At the end of the treatment period, the solid-liquid phase was released explosively into an expansion tank. The expansion tank was equipped with a gas valve were surplus steam is released. Detailed information about the PSC-technology are available on the internet (https://biomethane-map.eu/fileadmin/Biomethane_Map_-_TDs/UPD_TD_pressure_swing_conditioning_ventury_-_signature_website.pdf, 27 September 2019). Six variants of PSC-treated cattle manure (combinations of 5 min, 30 min, 60 min; 160 °C, 190 °C) and one untreated reference were investigated regarding the methane yields in BMP tests in laboratory scale in triplicates each. AMPTS devices (Bioprocesscontrol, Lund, Sweden, temperature set on 39 ± 1 °C) were used. BMP tests were conducted in accordance with the VDI guideline 4630 (2006). The methane yields were standardized (dry gas, 273.15 K, 1013.25 hPa).

Approximately 20 g of PSC-treated cattle manure and 400 g inoculum were weighted in for BMP-test. As inoculum served digestate gained from DBFZ’s research biogas plant (fed with cattle manure and corn silage) diluted with tap water 50% w/w.

### 2.2. Semi-Continuous Anaerobic Digestion Tests

Based on the results of the BMP tests, one PSC treatment variant of cattle manure (190 °C/30 min) and the reference were selected for a semi-continuous AD test in CSTR in laboratory scale with duplicates per variant of treatment. The ‘untreated’ cattle manure had to be chopped with a cutting mill (Fritsch Pulverisette 19/mesh size 6 mm) (Fritsch GmbH, Idar-Oberstein, Germany) in order to make the supply of the fermenters and the removal of digestate feasible (Supplementary Materials).

The semi-continuous AD tests with cattle manure were carried out in four CSTRs each with a net volume of 10 L (Bräutigam Kunststofftechnik GmbH, Mohlsdorf-Teichwolframsdorf, Germany). The temperature was set at 39 °C with a thermostat (JULABO GmbH, Seelbach, Germany). Stirrer ‘RZR 2102 control’ (Heidolph Instruments GmbH & Co.KG, Schwabach, Germany) were used at 50 rpm to mix the digesters. The biogas volume was measured with drum-type gas meter TG05/5 (Dr.-Ing. RITTER Apparatebau GmbH & Co. KG, Bochum, Germany). The biogas quality was determined with AwiFLEX No 1185_11 (Awite Bioenergie GmbH, Langenbach, Germany). CSTR tests were conducted in accordance with the VDI guideline 4630 (2006) [12] as well. Methane and biogas yields were standardized respectively (dry gas, 273.15 K, 1013.25 hPa).

The general feeding regime for semi-continuous AD tests at DBFZ is given in Liebetrau et al. (2016, p. 162) [14]. Sieved digestate (mesh size 4 mm, pH-value = 7.46, total solids (TS) = 7.69% FM, volatile solids (VS) = 77.35% TS, total ammonia nitrogen (TAN) = 1.49 g/L) of a full-scale biogas plant (DBFZ research biogas plant, Germany, was used as inoculum for the four lab-scale fermenters. The biogas plant was supplied with cattle manure and corn silage. The set temperature in the lab reactors was 39 ± 1 °C. After 10 days without feeding, the supply of all reactors with cellulose and pellets of DDGS (Distillers’ Dried Grains with Solubles) started due to a delay in the supply with cattle manure. At day 18 the feeding of all reactors with chopped cattle manure started and the organic loading rate (OLR) was raised to 2.5 g VS/(L·d) step by step. The hydraulic retention time (HRT) of 30 days was kept constant over the entire test. At the beginning (18th day) of the test 100 g fresh manure and 225 g tap water were fed daily to the reactors to adjust the ORL and HRT.

At day 57 the feeding of two reactors with PSC treated manure began, while the other two reactors remained as an untreated reference. In contrast to the reference manure, the PSC-manure was not chopped. Due to the fact that the dry matter content of the first PSC-material was a little bit lower than expected, the OLR of all four reactors had to be decreased from 2.5 to 2.4 g VS/(L·d). Hence, at day 57 two reactors were supplied with 333 g FM of PSC manure and the other two reactors with 128 g of reference manure plus 205 g tap water. In the course of the three HRT three material changes were necessary.

No additives like trace elements were used. The untreated manure and the PSC manure were stored in plastic barrels in a cooling chamber at 5 °C during laboratory tests.

### 2.3. Analytical Methods

Total solids (TS) and volatile solids (VS) were measured in accordance with DIN EN 12880 (2001) [15] and DIN EN 12879 (2001) [16]. The pH-value was measured with a pH device 3310 (WTW Wissenschaftlich-Technische Werkstätten GmbH, Weilheim, Germany). The analysis of furfural were conducted as described in Schumacher et al. (2019) [13]. Volatile organic acids and total inorganic carbon (VOA/TIC) and total ammonia nitrogen (TAN) were determined as described in Liebetrau et al. (2016, pp. 32, 34) [14].

### 2.4. Kinetic Modelling

For kinetic modelling single first-order kinetics according to Angelidakit et al., 2009 [17] have been applied to evaluate methane production kinetics of BMP tests. Based on Equation (1) the specific methane potential S_BMP_ (mL/g VS) and single first-order reaction constant k (1/d) of the investigated substrates need to be adjusted to depict progression of the cumulative methane production S (mL/g VS) over time.

(1) $$S\left(t\right)=\mathrm{BMP}\cdot \left(1-e^{\left(-k\cdot t\right)}\right)$$

Model implementations as well as numeric parameter identification were realized in the software environment Matlab (The MathWorks, Inc., Torrance, CA, USA) as previously describe in Schumacher et al. (2019) [13]. The coefficient of determination R^2^ (−) was calculated for each BMP test to evaluate model efficiency.

### 2.5. Statistical Analysis

#### 2.5.1. Batch

The BMP test results (final values of methane yield after 29 days) were analyzed with the help of the software SPSS statistics Version 20 (IBM, Chicago, IL, USA). In order to determine whether there was a significant difference between the methane yield of untreated (reference) and pretreated cattle manure, final data were evaluated using a Welch’s analysis of variance (ANOVA) taking a confidence level of 95% into account. If differences existed, a post hoc test according to Games Howell (α = 0.05) was chosen, to identify where they occurred.

#### 2.5.2. Semi-Continuous Tests

Semi-continuous tests (CSTR) were divided into pre-phase and three hydraulic retention times. All phases were analyzed separately with ANOVA and post hoc test by means of SPSS statistics Version 20 (IBM, Chicago, IL, USA). Further details about the selection of statistical tests are described by Hofmann et al. (2016) [18]. In order to meet the requirements of variance analysis with regard to their applicability, the experimental data were checked for normal distribution using the preliminary significance tests (Kolmogrov–Smirnov and Shapiro–Wilk). Furthermore, the assumption of variance homogeneity was checked by means of a preliminary Levene test. If the criterion of variance homogeneity could not be met, the significance test was performed with the help of Welch’s ANOVA.

## 3. Results and Discussion

### 3.1. Biochemical Methane Potential (BMP) Testing and Kinetic Modeling

Table 1 displays TS, VS, the final methane yields, and variation coefficients of BMP test after 29 days. The PSC pretreatment with 160 °C/60 min and 190 °C/30 min showed the highest methane yields with 239 mL/g VS and 229 mL/g VS, respectively. Budde et al. (2014) [7] published methane yields for untreated manure of at least 162 mL/g OM in BMP tests (30 days) and up to 255 mL/g OM (160 °C/5 min) for cattle manure pretreated with thermal pressure hydrolysis. Furthermore, Budde et al. (2014) [7] more often stated results between 216 and 232 mLCH_4_/g OM for treated manure. In this study the results for PSC treated manure are comparable to the data of Budde et al. (2014) [7], but the untreated manure has lower methane yields. In contrast Risberg et al. (2013) [6] found no improved gas production after pretreatment. Our study presents an increase by up to 109%, Table 1.

Possible causes for divergent results in various studies are: (a) substrate-specific: divergent physical and chemical composition; (b) pretreatment technology-specific: temperature set, pretreatment duration, the heating system (electrical jacket heating or with steam inside of the pressure tank), the chosen relaxation phase (gaseous or liquid), the heating speed and relaxation speed (cooling down slowly or explosive release) (c) BMP test-specific: microbiological consortium of the utilized inoculum, the temperature and the implementation of mixing, see also Schumacher et al. (2019) [13].

**Figure 1:**
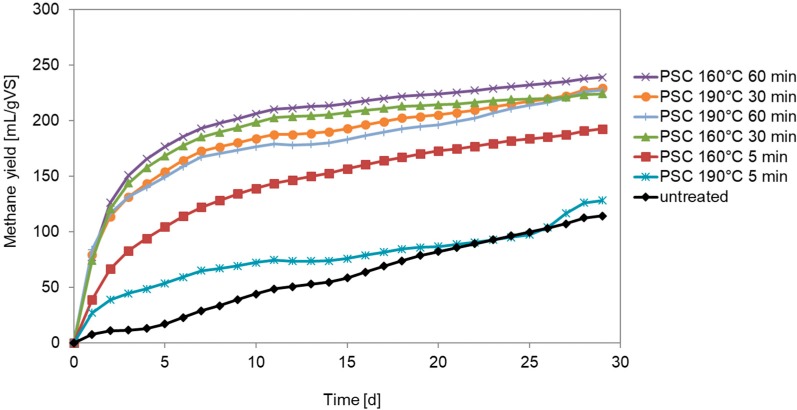
Methane yields in BMP tests.

The tremendous enhancement of degradation speed through PSC is visualized in Figure 1. Due to time limitations within the project the test termination criterion (daily biogas rate is equivalent to only 1% of the total volume of biogas produced up to that time, VDI 4630 2006 [12] for the untreated manure was not reached, see Figure 1.

To investigate degradation kinetics in more detail a first-order model was applied to depict experimental data. To ensure realistic simulation results the unknown first-order kinetic constants were estimated for fixed biochemical methane potentials. Thus, the final experimental value of the cumulative methane production after 29 days equals the biochemical methane potential S_BMP_ in the applied model structure (Equation (1)). As shown in Table 1 the resulting kinetic constant during pretreatment increases in comparison to the untreated sample. Furthermore, increasing intensity (length) of pretreatment from 5 to 30 or 60 min clearly shows an additional benefit in faster degradation kinetics during batch operation.

The statistical evaluation of the BMP results, using Welch’s ANOVA, delivered a significant result (*p* < 0.000). This means, the null hypothesis ‘There is no significant difference between the methane yield of untreated and PSC treated cattle manure’ can be rejected at a confidence level of 95%. In addition, the results from the Games–Howell post hoc test given under Table 2, show significant effects (*p* < 0.05) between the methane yield of control and the parameter combinations b, c, e and f.

However, the 68% or 12% increases in methane yield, associated with the PSC variants 160 °C/5 min and 190 °C/5 min, could not be confirmed statistically significant (*p* > 0.05).

### 3.2. Furfural Concentrations (BMP)

The following Table 3 shows the furfural concentrations of the liquid phase of pretreated cattle manure and the corresponding severity factor R_0_.

In all cases, the determined furfural concentrations were showing a relatively low level. The lowest value amounted to 2 mg/L whereas the highest concentration of 8.15 mg/L was corresponding with a severity factor R_0_ 4.43. Due to an insufficient amount of hydrolysate, it was not possible to determine the furfural concentration of the PSC variant 160 °C/60 min.

It is interesting to note that the release of furfural seems to correlate with the severity factor. Thus, a continuous increase of furfural concentration can be seen both over the pretreatment period and temperature. The latter seems to affect the severity (R_0_) and thus the formation of the inhibitor in a particular degree. For comparison: At the end of the PSC pretreatment at 160 °C, an increase of 85.5% of furfural was observed after 30 min compared to the five minutes, whereas the furfural concentration at 190 °C increased by about 180% from 5 min to 30 min sample.

In comparison to other publications, dealing with inhibitory effects of toxic compounds, an inhibition of the biogas process can probably be ruled out. As an example: Pekařová et al. (2017) [11] reported an inhibitory effect only at a furfural concentration between 1–2 g/L by using sodium acetate as a carbon substrate. By contrast, concentrations below 1 g/L seemed to have a stimulating effect on methane production. The inhibition was manifested through an increase in lag phase but after a certain time methane production was nearly restored with a comparable production rate to the control group.

Similar results were provided by Barakat et al., who also found no inhibition of the fermenter biology using furfural and 5-hydroxymethylfurfural (5-HMF) as single substrates (c = 2 g/L). The fermentation with 5-HMF showed a methane yield of 450 mL/g VS, whereas the digestion of furfural was 430 mL/g VS. The final level of methane production was reached after 10 days for furfural and 14 days for 5-HMF (Barakat et al., 2012) [8]. Both substances were initially associated with delays in methane production, which was explained by the prior conversion of the inhibitors to furfural alcohol (Barakat et al., 2012) [8] and (Rivard and Grohmann, 1991) [19].

### 3.3. Semi-Continuous Anaerobic Digestion Tests

#### 3.3.1. Methane Production

On the base of the BMP test, the PSC-variant 190 °C/30 min (variation coefficient lower than variant 160 °C/60 min) was chosen for the semi-continuous fermentation test. A positive effect of PSC treatment on the methane production was observed at the beginning of the first HRT until the first change of PSC treated substrate. During the second HRT the methane production of PSC manure decreased to the reference level, while the variance of the methane production of PSC manure is visibly higher than the variance of the reference. After the third material change and at the beginning of the third HRT the methane production of PSC manure decreased below the level of the reference. During the test period new substrate had to be collected and treated due to limited cooling capacity and the lack of stability of the substrate. This seems to cause a varying composition and/or structure of the treated substrate at different times, which could be a reason for varying methane production. The average methane production of PSC-treated manure was 239 mL/g VS from the beginning of feeding with treated substrate until the end of the semi-continuous fermentation test and 235 mL/g VS of the reference, respectively.

##### Statistical Tests

**Figure 2:**
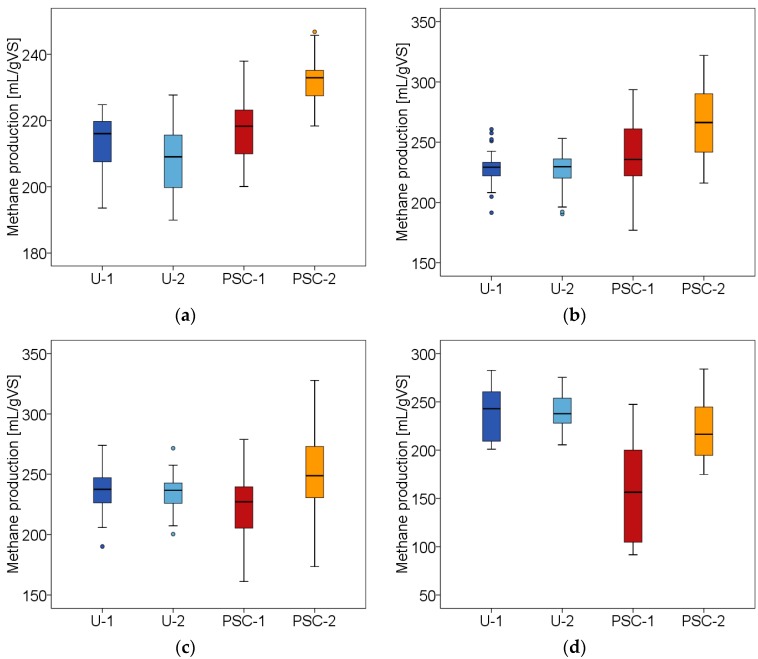
Boxplots of methane production (**a**) pre-phase; (**b**) hydraulic retention time (HRT) 1; (**c**) HRT 2; (**d**) HRT 3.

For the statistical evaluation of the semi-continuous test period, the distribution of the measured methane production is illustrated with the help of boxplots. This allows presumptions on the result of every single phase and on the fulfillment of the assumptions for statistical tests according to Hofmann et al. (2016) [18]. Figure 2 shows the boxplots of the biogas yields for the pre-phase and three following hydraulic retention times. The boxplots displays four reactors where two are assigned to the reference (U = untreated) and two to the PSC treatment. The median of the measurement data is represented by the band in the middle of the box and is limited by the first and third quartiles. Furthermore the whiskers, are illustrated with 1.5 times the interquartile distance (IQR), which can be used to approximate the distribution of the variances. The variances can be considered homogeneous, if whiskers have an equal length. In addition, outliers can be displayed. A distinction is made between mild and extreme outliers. Mild outliers have a distance to the first or third quartile of 1.5× IQR to 3.0× IQR. In a SPSS box plot, these values are marked with individual dots.

##### Pre-Phase

The boxplot in Figure 2a illustrates the experimental data of the pre-phase where no PSC-treatment of cattle manure occurred. It is expected, that neither the methane production between the reactors nor between the approaches will differ in this phase. However, this expectation seems to be refuted by the first visual impression. Instead, boxplot shows a slight difference between the methane yields between the two approaches. With the help of ANOVA, the first impression was confirmed (*p* = 0.000) meaning, the null hypothesis ‘There is no significant difference in methane yield between the approaches’ had to be rejected. To identify where the differences occurred, a Bonferroni post-hoc test was used. The test procedure confirmed that reactor PSC-2 significantly differed from U-1 (*p* = 0.000), U-2 (*p* = 0.000) and PSC-1 (*p* = 0.000). Furthermore, a significant difference between the methane yields of the second reactor of the control group U-2 (*p* = 0.015) compared to the methane yield of the first reactor of the PSC approach, PSC-1 could be revealed. A possible reason for these deviations could be that most digesters had not reached stable conditions by the end of the pre-phase. A regression analysis showed trends of the methane yield over time in three out of four cases which questions the assumption of steady-state conditions.

##### Hydraulic Retention Time (HRT) 1

As already mentioned, the positive effect of PSC-treatment on methane production could only be observed at the beginning of the first hydraulic retention time until the first change of pretreated material. Afterwards, the methane value of both PSC-reactors clearly decreased. This result is reflected in Figure 2b. Regarding the boxplots, only small effects on the methane production by the PSC-treatment can be observed. In order to assess differences between the methane productions of reference- and PSC-approaches, methane values were considered over the entire period. As the Levene-statistics indicate heterogeneity of variances a Welch’s ANOVA was used for the statistical evaluation. Comparing the methane production of the pre-treatment related to the reference a significant difference (*p* = 0.000) between the two approaches could be observed. Nevertheless, the results must be called into question at this point. On the one hand a following Games–Howell test showed that the difference primarily depends on PSC-2 while no significant differences between PSC-1 and U-1 (*p* = 0.211) or U-2 (*p* = 0.094) could be determined. On the other hand, a regression analysis revealed a trend over time which also indicates that steady state conditions were not reached.

In order to make a statement about the influence of PSC-approach, the effect of the substrate disintegration adjusted for the effect of time, was determined by means of linear regression taking a confidence level of 95% into account (Table 4).

Here, the regression coefficient ‘Measuring time’ represents the influence of the time factor on the methane production of the PSC approach. If the influence of the disintegration approach is adjusted for this value, a statement about the pure effect of the disintegration can be made. Thus, the analysis showed that methane production increased by an average of 25.733 mL/g VS compared to the reference approach.

Nonetheless, the influence of the time factor cannot be ignored in the final assessment. Moreover, possible interaction effects between disintegration approach and time should not be disregarded. Therefore, in a second regression analysis it was examined whether there is an interaction effect between the time of measurement and the approach and to what extent this influences the methane production of the PSC approach (Table 5). 

The analysis showed significant interaction effect between measuring time and the methane production of the PSC-approach at *p* = 0.000. A regression coefficient of −3.110 indicates a decreasing effect over time. In other words, the increase in methane yield from 25.733 mL/g VS could not be maintained until the end of the first retention time.

##### HRT 2

Nearly no trend over time and, therefore, steady-state conditions were confirmed for the methane production of all digesters given in Figure 2c. The box plots do not show any differences between the methane productions of the approaches. As the Levene-statistics indicate heterogeneity of variances a Welch’s ANOVA verified the first impression by retaining the null hypothesis of ‘There is no significant difference between the methane production of different approaches’ at *p* = 0.907. Only a slight deviation is indicated within the PSC-approach. A further Welch’s ANOVA delivered a significant difference between PSC-1 and PSC-2 at *p* = 0.040. However, the positive effect of disintegration on the methane production which could still be observed at the beginning of pre-treatment is no longer discernible towards the end of the first hydraulic retention time.

##### HRT 3

**Figure 3:**
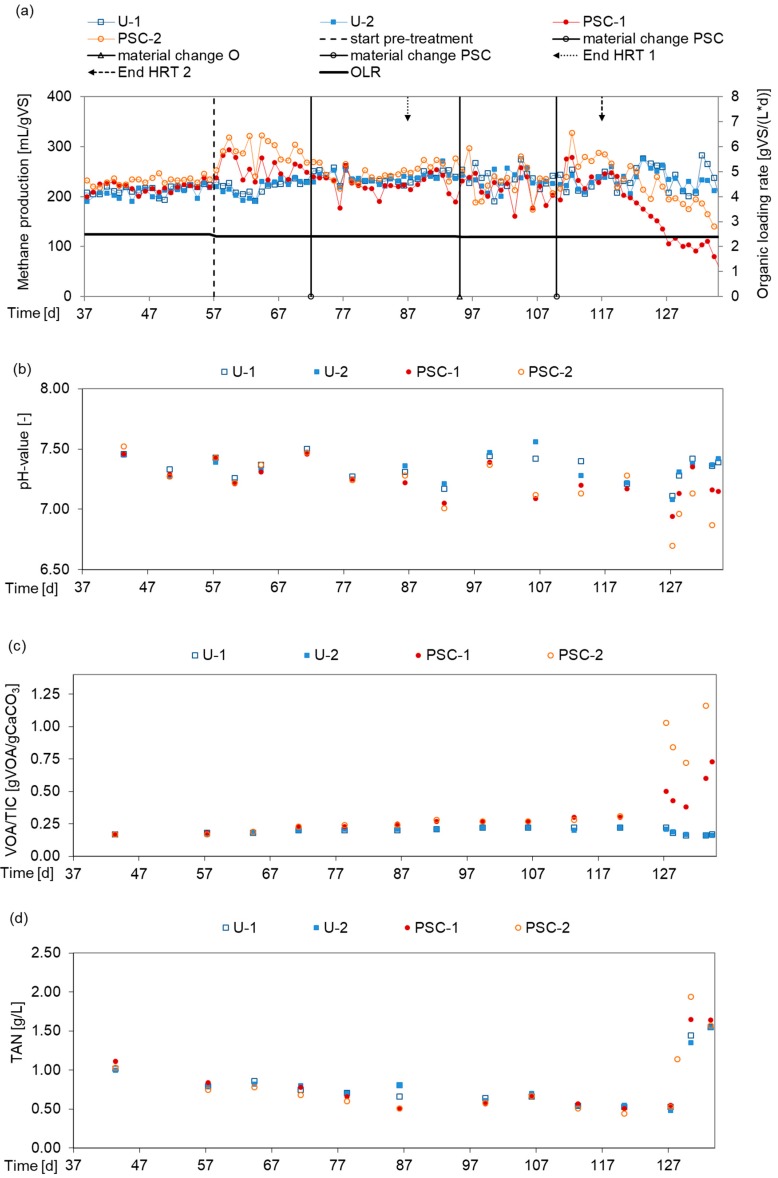
(**a**) Methane production of cattle manure in semi-continuously stirred tank reactor (CSTR) (U = untreated, PSC = pressure swing conditioning); (**b**) pH-value; (**c**) volatile organic acids and total inorganic carbon (VOA/TIC); (**d**) total ammonia nitrogen (TAN).

The final results the third hydraulic retention time are shown in Figure 2d. Taking the regression analysis into account, stationary conditions in methane production could only be determined for the reactors of the reference approach, whereas the fermenters, which were fed with pre-treated cattle manure, showed a clear decrease in the methane production indicating a trend over time. Since the Levene test indicated a heterogeneous distribution of the experimental data, the methane production of both the approaches and the reactors were statistically examined with the help of Welch’s ANOVA. In both cases, the analysis revealed a significant result at *p* = 0.000. Considering the course of methane production shown in Figure 3a this result was expected. The following Games–Howell post-hoc test showed that the difference between the approaches was due to PSC-1, which differed significantly from PSC-2 (*p* = 0.006), U-1 (*p* = 0.000) and U-2 (*p* = 0.000), respectively. The positive influence of the pre-treatment, which had already disappeared towards the end of the first hydraulic retention time, could no longer be demonstrated at the end of the test.

#### 3.3.2. pH-Value, Volatile Organic Acids and Total Inorganic Carbon (VOA/TIC) and Total Ammonia Nitrogen (TAN)

Figure 3a shows methane production of the duplicates for the PSC variant and the reference over the pre-phase and the 3 HRT. At the 127th day the pH-value dropped from 7.17 to 6.94 and from 7.28 to 6.70 in the PSC digestate, Figure 3b. The reference’s digestate decreased less sharply from 7.22 to 7.08 and 7.21 to 7.11 between the days 120 to 127, Figure 3b. At the same time, the VOA (6.59 g/L) and VOA/TIC (1.16 gVOA/gCaCO_3_) strongly increased particularly in reactor PSC-2, Figure 3c. During the test the TAN constantly decreased in the PSC reactors and in the references, Figure 3d. At 127th day 40 g of ammonium hydrogen carbonate were added to avoid acidification, but at day 134/135 the PSC reactors had to be finished due to the acidification.

### 3.4. Comparison of BMP and Semi-Continuous Anaerobic Digestion Tests

The methane yields of PSC manure in BMP and the average in semi-continuous AD were comparable, but the reference showed strong differences in methane yields. A reason for this was the chopping of the manure with a cutting mill (mesh size 6 mm) for the semi-continuous test, to facilitate feeding and sampling of the digesters in contrast to the uncrushed manure in BMP. 

### 3.5. Outlook

The statistical analyses revealed that it would be advisable to check the steady-state conditions and the variances of methane production between the reactors in the pre-phase before changing to pre-treated substrate in semi-continuous tests. The storage conditions of manure and the treatment frequency will always influence the results. An optimized combination of temperature/treatment duration for PSC, the observation of effects of PSC on microbiological community, improved supply with nitrogen and trace elements might be a starting point for further investigations with the objective of stable methane production in (semi-)continuous AD-tests at lab-scale or full-scale.

## 4. Conclusions

During the BMP test of PSC treatment of cattle manure, the methane yield increased by a minimum 12% and maximum 109% compared to the untreated reference; 160 °C/60 min and 190 °C/30 min showed the highest methane yields. The degradation was accelerated by PSC treatment and the increase of the methane yield after 29 days statistically significant, if the treatment duration was 30 or 60 min for both chosen temperatures (160 °C, 190 °C).

The methane yields of PSC manure in BMP and the average in semi-continuous AD were at the same level. The reference showed strong differences in methane yields because of extra chopping of the manure with a cutting mill for the semi-continuous AD, to facilitate feeding and sampling of the digesters. In semi-continuous AD tests in lab-scale CSTR the methane production of PSC treated cattle manure increased only temporarily during the first HRT, but a positive effect of PSC in steady state could not be proven.

However, for stable continuous processes substrate-specific parameter sets or ranges for PSC have to be determined and applied. Also, the framework conditions like nitrogen and trace element supply have to be optimal. It is presumable that the enhancement of the methane yields through PSC under full-scale conditions is higher than under lab-scale conditions because of the extra mechanical treatment of the reference. However, PSC has the potential to facilitate the handling, enhance degradation speed and, at best, to increase the methane yield of ligno-cellulosic substrates in full-scale biogas plants.

## Figures and Tables

**Table 1:** Final methane yields of untreated and pressure-swing conditioning (PSC)-treated cattle manure (biochemical methane potential (BMP) test) including total solids (TS), volatile solids (VS), variation coefficient, increase of methane yield, first-order kinetic constant and coefficient of determination.

	Total Solids	Volatile Solids	Methane Yield	Variation Coefficient	Increase of CH_4_ Yield	First-Order Kinetic Model ^a^	
						k	R^2^
	%FM	%TS	[mL/g VS]	[−]	[%]	[1/d]	[−]
untreated	20.87	75.52	114	0.01	-	0.0594	0.92
PSC 160 °C 5 min	13.24	81.43	193	0.07	68.43	0.1339	0.96
PSC 160 °C 30 min	13.18	84.72	224	0.01	96.11	0.2822	0.95
PSC 160 °C 60 min	16.09	84.76	239	0.22	109.20	0.2591	0.92
PSC 190 °C 5 min	13.16	73.39	128	0.07	12.16	0.0723	0.81
PSC 190 °C 30 min	10.36	79.50	229	0.04	100.53	0.1916	0.85
PSC 190 °C 60 min	8.00	79.55	227	0.02	98.94	0.1714	0.76

^a^ Estimated for fixed methane potentials S_BMP_ determined during BMP tests (final experimental methane yield) after 29 days.

**Table 2:** Significance test for BMP.

Control	Parameter Combination	Mean Difference [mL/g VS]	Significance
untreated	a: PSC 160 °C 5 min	−78.29	0.195
	b: PSC 160 °C 30 min	−109.87 *	0.002
	c: PSC 160 °C 60 min	−124.60 *	0.001
	d: PSC 190 °C 5 min	−13.93	0.836
	e: PSC 190 °C 30 min	−115.12 *	0.049
	f: PSC 190 °C 60 Min	−113.13 *	0.013

* Significant difference between the methane yield reference and pretreated cattle manure (confidence level of 95%).

**Table 3:** Furfural concentrations of pretreated cattle manure and severity factor.

	Furfural Concentration [mg/L]	Severity Factor, log R_0_ [−]
PSC 160 °C 5 min	2.00	2.47
PSC 160 °C 30 min	3.71	3.24
PSC 160 °C 60 min	-	3.54
PSC 190 °C 5 min	2.08	3.35
PSC 190 °C 30 min	5.83	4.13
PSC 190 °C 60 min	8.15	4.43

**Table 4:** Linear regression for substrate disintegration adjusted for the effect of time.

	Non-Standard Coefficients		Standard		
	Regression Coeficient	Standard Error	Coefficient Beta	T	Significance
(Constant)	211.371	8.076		26.172	0.000
Measuring Time	−0.621	0.257	−0.193	−2.417	0.017
PSC-Approach	25.733	4.445	0.463	5.790	0.000

**Table 5:** Linear regression to determine the interaction effect between PSC-approach and time.

	Non-Standard Coefficients		Standard		
	Regression Coeficient	Standard Error	Coefficient Beta	T	Significance
(constant)	138.600	11.959		11.589	0.000
Measuring time	4.074	0.674	1.269	6.048	0.000
PSC-approach	74.247	7.564	1.336	9.816	0.000
Interaction (time/PSC-approach)	−3.310	0.426	−1.771	−7.346	0.000

## Supplementary Materials

The following are available online at https://www.mdpi.com/2306-5354/7/1/6/s1: Figure S1: Untreated for batch test; PCS-treated for batch /continuous tests, ‘Untreated’ chopped for continuous test, f.l.t.r. (Source: DBFZ).
