# Supplementary material for: The Influence of Pressure-Swing Conditioning Pre-Treatment of Cattle Manure on Methane Production
Source: Bioengineering (Basel). 2019 Dec 30;7(1):6. doi: 10.3390/bioengineering7010006 (PMC7175210; doi:10.3390/bioengineering7010006)
Supplement: Supplementary file 1 [file bioengineering-07-00006-s001.pdf]

Supplementary material

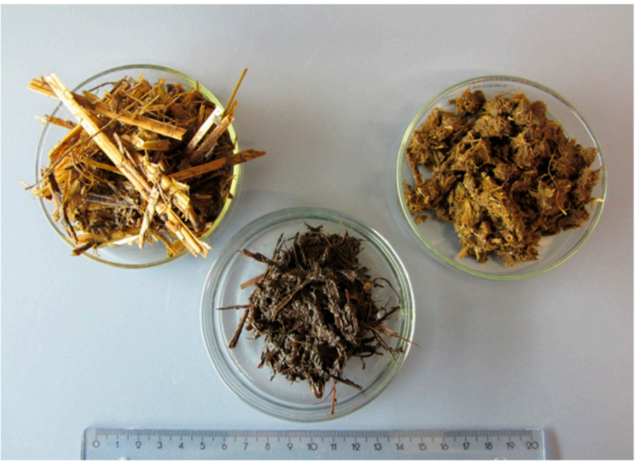

Figure S1. Untreated for batch test; PCS-treated for batch /continuous tests, 'Untreated' chopped for continuous test, f.l.t.r. (Source: DBFZ).
